# Statins and Fibrates in Age-Related Macular Degeneration: A Contemporary Clinical Narrative Review (2020–2025)

**DOI:** 10.3390/jcm15082960

**Published:** 2026-04-14

**Authors:** Weronika Dmoch, Julia Sawicka, Natalia Żelichowska, Zuzanna Kępczyńska, Piotr Maciejewicz, Dariusz Kęcik

**Affiliations:** 1 Ophthalmology Student Research Group, Department of Ophthalmology, Medical University of Warsaw, 4 Lindleya Street, 02-005 Warsaw, Poland; s082689@student.wum.edu.pl (J.S.); s080347@student.wum.edu.pl (N.Ż.); zuzanna.kepczynska@pentahospitals.pl (Z.K.); 2 Department and Clinic of Ophthalmology, University Clinical Center, Medical University of Warsaw, 4 Lindleya Street, 02-005 Warsaw, Poland; piotr.maciejewicz@wum.edu.pl (P.M.); dariusz.kecik@wum.edu.pl (D.K.)

**Keywords:** age-related macular degeneration, statins, fenofibrate, hydroxymethylglutaryl-CoA reductase inhibitors, oxidative stress, inflammation, dyslipidemias, retinal drusen, choroidal neovascularization, geographic atrophy

## Abstract

Age-related macular degeneration (AMD) remains the leading cause of irreversible central vision loss in the elderly. Increasing attention has been directed toward lipid metabolism as a potential contributor to disease onset and progression. The overlap between AMD and atherosclerosis—particularly regarding lipid accumulation, endothelial dysfunction, and chronic inflammation—has prompted interest in lipid-lowering therapies. This narrative review synthesizes the clinical evidence published between 2020 and 2025 on the potential role of statins and fenofibrate in AMD risk modification and disease progression. A structured literature search was conducted in PubMed, Scopus, and Web of Science using combined MeSH and free-text terms related to lipid-lowering agents and AMD. Human studies evaluating clinical incidence or progression outcomes were considered alongside contextual evidence from prior evidence syntheses. Overall, findings remain heterogeneous. Most studies did not demonstrate a consistent association between statin therapy and AMD incidence or progression in unselected populations. However, selected reports suggested a potential delay in dry AMD onset or slower disease progression among patients receiving prolonged or higher-intensity statin treatment. Evidence regarding fenofibrate was more limited and heterogeneous, with only a tentative protective signal observed in adherent users, particularly for non-exudative AMD. The current literature does not support lipid-lowering therapy as a universal preventive strategy for AMD. Nonetheless, subgroup-specific benefits cannot be excluded, especially in early disease stages or metabolically high-risk populations. Further well-designed prospective and randomized studies are needed to clarify therapeutic relevance and identify the patients who are most likely to benefit.

## 1. Introduction

Age-related macular degeneration (AMD) is a chronic, progressive retinal disorder and the leading cause of irreversible central vision loss among older adults worldwide [1,2,3]. The disease profoundly affects the quality of life by impairing essential daily functions such as reading, driving, and facial recognition [4]. With global population aging, the prevalence of AMD continues to rise, intensifying the need to identify modifiable risk factors and preventive strategies [5,6].

The pathophysiology of AMD is multifactorial and involves a complex interplay of genetic susceptibility, oxidative stress, lipid dysregulation, inflammation, and age-related structural alterations within the retina and choroid [7,8,9]. The accumulation of lipid-rich deposits, particularly drusen, between the retinal pigment epithelium (RPE) and Bruch’s membrane (BrM) initiates degenerative cascades including local inflammatory activation and neovascular remodeling [8,10,11]. Notably, these lipid-driven processes share mechanistic parallels with atherosclerosis, encompassing endothelial dysfunction, lipid peroxidation, and chronic immune activation [6,8,12].

**Figure 1:**
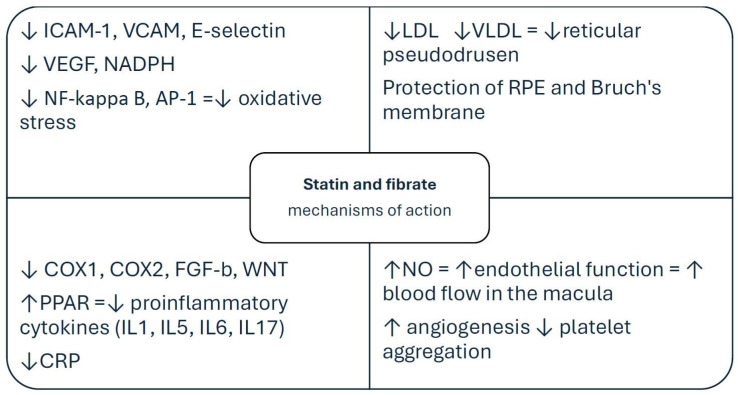
The proposed mechanisms by which statins and fenofibrates may influence AMD pathogenesis, including lipid-lowering, anti-inflammatory, antioxidant, endothelial, and anti-angiogenic effects. Some pathways illustrated in the figure are supported by ocular or retinal biologic plausibility, whereas others remain extrapolated primarily from experimental, translational, or cardiovascular models and have not been directly confirmed in human retinal tissue in the context of AMD. Upward and downward arrows indicate increase and decrease, respectively.

Given these biological overlaps, lipid-lowering therapies—especially statins and fibrates—have emerged as potential modulators of AMD risk and progression. Statins, as hydroxymethylglutaryl-CoA reductase inhibitors, not only reduce systemic cholesterol levels but also exert pleiotropic effects, including anti-inflammatory, antioxidant, and anti-angiogenic actions [13,14,15,16,17]. Fibrates, acting primarily through peroxisome proliferator-activated receptor alpha (PPAR-α) activation, influence lipid metabolism and inflammatory signaling pathways that may also be relevant to AMD pathogenesis (Figure 1) [8,18,19,20].

Despite the compelling mechanistic rationale, the clinical findings remain heterogeneous. Some studies suggest a reduced risk of AMD onset or slower disease progression among the individuals receiving long-term or intensive statin therapy, whereas others demonstrate no significant association [13,14,15,21,22,23,24]. The interest in fibrates has been fueled by their protective effects observed in diabetic retinopathy; however, their role in AMD prevention or modification remains insufficiently defined [13,18].

This narrative review synthesizes recent evidence published between 2020 and 2025 to clarify the potential impact of statins and fibrates on AMD incidence and progression. By integrating findings from clinical studies and prior evidence syntheses, we aim to contextualize therapeutic signals, identify areas of inconsistency, and highlight priorities for future research.

## 2. Methods

This narrative review was conducted using a structured literature search strategy to identify and synthesize recent evidence on lipid-lowering therapies in age-related macular degeneration (AMD).

A literature search was performed in PubMed (National Library of Medicine, Bethesda, MD, USA), Web of Science (Clarivate, London, UK), and Scopus (Elsevier B.V., Amsterdam, The Netherlands) on 9 January 2025. The search strategies combined MeSH and free-text terms related to lipid-lowering agents and AMD, including “statins,” “hydroxymethylglutaryl-CoA reductase inhibitors,” “fenofibrate,” “fibrates,” and “age-related macular degeneration,” using Boolean operators (AND, OR) as appropriate. An example search strategy used in PubMed was: (“statins” OR “hydroxymethylglutaryl-CoA reductase inhibitors” OR “fenofibrate” OR “fibrates”) AND (“age-related macular degeneration” OR “AMD”).

The studies published between 2020 and 2025 in English and involving human participants were considered. The 2020–2025 time window was applied systematically to clinical studies evaluating AMD outcomes in relation to lipid-lowering therapy, whereas earlier and more recent references were included in a non-systematic manner to provide a mechanistic, epidemiological, and health-economic context.

The inclusion criteria comprised randomized controlled trials, cohort studies, and case–control studies evaluating the associations between lipid-lowering therapy and AMD incidence, progression, or visual outcomes. The exclusion criteria included non-human studies, editorials, case reports, narrative reviews without original clinical data, and studies lacking relevant exposure or outcome measures. In addition, the selected systematic reviews and meta-analyses were consulted to contextualize the clinical findings and to provide a broader interpretative framework that is consistent with the narrative review methodology.

The records retrieved from the databases were exported and screened in two stages by two independent reviewers. First, the duplicate records were identified and removed. The titles and abstracts of the remaining studies were screened for relevance to AMD and lipid-lowering therapy. The studies that were clearly unrelated to the topic, non-human studies, editorials, or publications without original clinical data were excluded at this stage. Full-text versions of potentially eligible articles were then assessed for eligibility based on predefined criteria, including study design, relevance of outcomes, and the availability of data on statin or fibrate exposure. The discrepancies were resolved through discussion.

**Figure 2:**
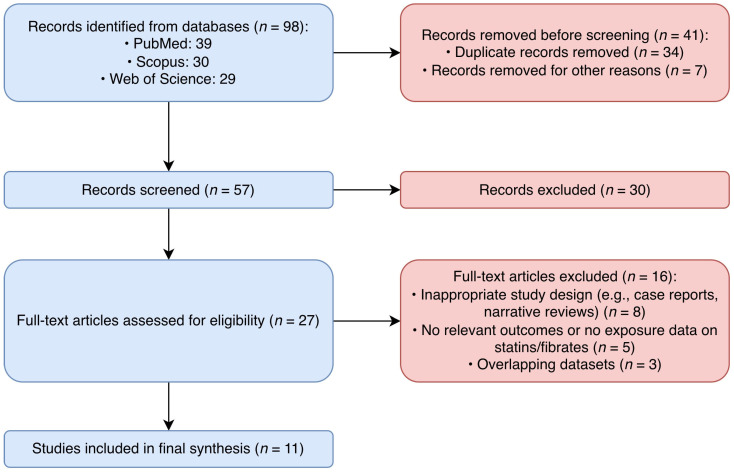
A flow diagram of the study selection process.

In total, 98 records were identified from the database searches (PubMed: 39; Scopus: 30; Web of Science: 29). After the removal of 34 duplicate records and 7 records removed for other reasons (e.g., non-human studies or publication type), 57 titles and abstracts were screened. Thirty records were excluded during title and abstract screening due to a lack of relevance to AMD or lipid-lowering therapy. The full texts of 27 articles were assessed for eligibility, of which 16 were excluded for the following reasons: an inappropriate study design (e.g., case reports or narrative reviews) (*n* = 8), a lack of relevant outcomes or an absence of data on statins or fenofibrate (*n* = 5) and overlapping datasets with other included studies (*n* = 3). Ultimately, 11 studies were included in the final synthesis. The study selection process is summarized in Figure 2.

The data from the included studies were extracted descriptively, focusing on study design, geographic setting, population characteristics, AMD subtype, exposure definition (drug class, dosage, and duration), and reported ophthalmic outcomes.

The methodological quality was assessed narratively. No formal standardized risk-of-bias tool was applied, given the narrative design of the review; however, key domains of methodological rigor were systematically considered, including study design, adequacy of confounding control, clarity of exposure definition, reliability of AMD diagnosis, and completeness of outcome reporting. Only studies directly evaluating the association between lipid-lowering therapy and AMD-related clinical outcomes were considered eligible for inclusion. Due to the heterogeneity in study design, treatment definitions, and outcome reporting, findings were synthesized qualitatively rather than quantitatively.

The outcomes of interest included AMD incidence, disease progression, visual acuity changes, and structural retinal biomarkers, including drusen burden, where available. No new human or animal research was conducted; therefore, ethical approval was not required.

## 3. Clinical Evidence on Lipid-Lowering Therapy in AMD

### 3.1. Overview of Available Clinical Studies

The recent literature examining the relationship between lipid-lowering therapies and age-related macular degeneration (AMD) comprises a heterogeneous body of clinical evidence published between 2020 and 2025. The available studies include large population-based cohort analyses, case–control investigations, and secondary evidence syntheses conducted across diverse geographic regions, including the United States, Taiwan, Iran, Canada, Cyprus, Greece, and Romania (Table 1).

Collectively, these studies represent the data derived from more than five million participants, with individual sample sizes ranging from several thousand to over two million individuals. The predominance of observational designs reflects the limited availability of randomized controlled trials evaluating lipid-lowering therapy in AMD. The study populations varied substantially with regard to the baseline cardiovascular risk, metabolic comorbidities, AMD subtype distribution, and treatment exposure definitions.

Across the body of evidence, reported associations between lipid-lowering therapy and AMD outcomes were inconsistent. Most analyses suggested no statistically significant relationship between statin or fibrate exposure and AMD incidence or progression in the unselected populations. However, several studies indicated potential protective trends within specific clinical contexts, particularly among patients receiving long-term or high-intensity therapy.

Given the variability in study design, exposure definitions, outcome classification, and follow-up duration, findings were interpreted qualitatively. The differences in AMD staging criteria, treatment adherence, cumulative dosing, and demographic structure likely contributed to heterogeneity in the reported results. Notably, the investigations focusing on early or intermediate AMD more frequently suggested potential therapeutic benefits than those evaluating advanced disease stages. One possible explanation is that lipid-modifying and anti-inflammatory interventions may be more biologically relevant in earlier disease phases, when drusen accumulation, retinal pigment epithelium stress, and subclinical vascular dysfunction are still potentially modifiable. In contrast, advanced geographic atrophy or neovascular AMD may reflect more structurally established disease, in which irreversible tissue damage or late-stage angiogenic remodeling limits the observable impact of systemic lipid-lowering therapy. This pattern should be interpreted cautiously, as differences in disease staging, exposure ascertainment, and baseline cardiovascular and metabolic risk may have influenced the apparent variability in treatment effects across studies.

### 3.2. Statins and Age-Related Macular Degeneration

The evidence evaluating the association between statin therapy and AMD risk remains mixed and inconclusive. Several high-quality meta-analyses and systematic reviews [5,15] reported no demonstrable effect of statins on AMD incidence or progression. Comparable neutral findings were observed in a large case–control study conducted among diabetic patients [21], as well as in a longitudinal cohort study by Ludwig et al. [10], which found no association between statin exposure and progression to exudative AMD.

The absence of consistent benefit has been further emphasized in broader evidence syntheses highlighting the limited number and heterogeneity of randomized controlled trials in this field [4,13]. These analyses underscore the methodological challenges that are inherent in isolating lipid-lowering effects from confounding cardiovascular and metabolic variables.

Importantly, much of the available clinical evidence derives from observational studies, which are inherently vulnerable to several sources of bias. One important consideration is confounding by indication, as patients receiving statin therapy often differ systematically from untreated individuals with respect to cardiovascular risk profiles, metabolic comorbidities, and healthcare utilization patterns. These differences may independently influence AMD risk and progression.

Additionally, studies based on administrative or prescription databases may be subject to a misclassification of medication exposure, as prescription records do not necessarily reflect actual treatment adherence or cumulative drug intake. The variability in the definition and diagnostic criteria of AMD across studies, particularly when based on registry data or diagnostic codes, may further contribute to inconsistent findings.

Conversely, selected studies have suggested potential protective associations under specific conditions. A meta-analysis by Mauschitz et al. [14] reported an inverse relationship between lipid-lowering therapy and AMD prevalence in European populations, although analyses did not differentiate between statins and fibrates. Ganesh et al. [9] described a delayed onset of dry AMD among statin users, while Chen et al. [6] identified a possible dose–response relationship, whereby lower statin exposure was associated with an increased AMD risk, and a higher cumulative dosing correlated with delayed disease progression and improved visual outcomes [4,6]. One possible explanation for this paradoxical pattern is that low-dose statin exposure may be insufficient to meaningfully influence retinal lipid handling, inflammatory activity, or endothelial dysfunction, whereas a higher cumulative exposure could be required to achieve biologically relevant pleiotropic effects. At the same time, this apparent contrast may also reflect non-causal factors, including differences in baseline cardiovascular risk, treatment indication, and adherence behavior between lower- and higher-exposure groups. Therefore, the observed low-dose/high-dose divergence should be interpreted as a hypothesis-generating signal rather than definitive evidence of a true biological threshold effect.

Importantly, the available literature does not apply uniform definitions of “long-term”, “intensive”, or “high cumulative exposure”, which limits the comparability across studies. Moreover, the apparent dose–response relationships described in some observational datasets may reflect residual confounding, differential healthcare utilization, or adherence-related bias rather than a true pharmacologic gradient.

Taken together, current evidence does not support a uniform protective role of statins in AMD prevention. Instead, the available literature suggests that any potential therapeutic signals may be restricted to specific subgroups rather than generalizable across all patients. These include individuals with early or intermediate AMD, patients with metabolic comorbidities such as dyslipidemia or diabetes, and those exposed to prolonged or higher cumulative statin treatment. However, at present, these subgroup-specific observations should be considered hypothesis-generating rather than definitive, as they derive predominantly from observational studies with heterogeneous exposure definitions and varying susceptibility to residual confounding.

### 3.3. Fibrates and Age-Related Macular Degeneration

Compared with statins, clinical evidence regarding fibrate therapy in AMD remains limited. The available data derive primarily from observational cohort analyses, with relatively few studies directly evaluating AMD-specific outcomes.

Two cohort investigations [10,18] reported no statistically significant association between fibrate exposure and AMD incidence or disease progression. These findings align with the broader uncertainty surrounding the ophthalmic impact of the fibrate-mediated lipid modulation outside of diabetic retinopathy contexts.

Nevertheless, subgroup observations suggest potential context-dependent effects. Wang et al. [18] reported that patients demonstrating high adherence to fibrate therapy exhibited a reduced risk of developing dry AMD, indicating that treatment compliance and cumulative exposure may influence retinal outcomes. Secondary evidence syntheses, including the meta-analysis by Mauschitz et al. [14], further support the hypothesis that lipid-lowering therapy as a broader pharmacologic category may confer modest protective effects against AMD development, although fibrate-specific conclusions remain constrained by limited and heterogeneous data.

Overall, the evidence base for fibrates is characterized by smaller study numbers, fewer adjusted analyses, and less consistent outcome reporting compared with statins. As such, definitive conclusions regarding their role in AMD prevention or progression cannot yet be established.

## 4. Discussion

Age-related macular degeneration (AMD) represents one of the leading causes of irreversible vision loss in aging populations and constitutes a major public health challenge due to its progressive nature and the socioeconomic burden associated with long-term management [25,26,27]. As therapeutic options remain limited, particularly in early disease stages, considerable attention has been directed toward modifiable systemic risk factors, including dyslipidemia. The systemic vascular and metabolic comorbidities, as well as pharmacological exposures, have therefore been increasingly investigated as potential modulators of AMD pathophysiology [8].

Accumulating evidence suggests that lipid metabolism plays a relevant role in AMD pathogenesis. Histopathological studies have identified basal laminar deposits, composed largely of lipoprotein-derived material, as a defining structural feature of the disease [6,28,29]. Similarly, drusen—lipid-rich extracellular deposits located between the retinal pigment epithelium and Bruch’s membrane—contain cholesterol and apolipoproteins, further supporting the mechanistic parallels between AMD and atherosclerotic processes [30,31,32]. These observations have prompted growing interest in lipid-lowering therapy as a potential modifier of disease onset and progression, particularly among patients with systemic hyperlipidemia [6,33].

Statins, as inhibitors of hydroxymethylglutaryl-CoA (HMG-CoA) reductase, exert their primary therapeutic effect through cholesterol reduction. However, their biological activity extends beyond lipid modulation. The experimental and clinical data indicate that statins may influence AMD-relevant pathways through anti-inflammatory, antioxidant, and endothelial-stabilizing effects [34,35]. Suppression of pro-inflammatory cytokines, attenuation of oxidative stress, and improved endothelial function may collectively enhance choroidal perfusion and retinal pigment epithelium integrity [36,37,38]. These pleiotropic mechanisms are particularly relevant in the context of AMD’s multifactorial pathophysiology.

The evidence regarding structural retinal outcomes remains limited but suggestive. Statin exposure has been associated with reductions in drusen burden and improvements in selected clinical parameters in certain patient cohorts [4]. Such findings provide a potential clinical correlation to the proposed anti-inflammatory and lipid-modulating effects of statins, although a direct causal relationship remains uncertain. Additional observations indicate that lipid-lowering therapy may modulate angiogenic signaling pathways, including the downregulation of the vascular endothelial growth factor (VEGF), which could have implications for neovascular AMD progression [39]. However, direct clinical evidence linking these molecular mechanisms with improved long-term AMD outcomes remains limited, and many mechanistic insights are still derived primarily from experimental or preclinical models. In particular, effects such as anti-inflammatory signaling, oxidative stress modulation, and endothelial stabilization have some biologic plausibility in ocular tissues, whereas pathways such as COX inhibition or WNT-related signaling should be interpreted more cautiously, as they are supported predominantly by extrapolation from experimental systems or broader vascular models rather than from direct confirmation in human retinal tissue.

Fibrates have also attracted attention as potential modulators of retinal disease. Through the activation of the peroxisome proliferator-activated receptor alpha (PPAR-α), these agents influence lipid metabolism, inflammatory cascades, and microvascular function [40,41]. The preclinical models suggest that fenofibrate may mitigate AMD-related processes by inhibiting cyclooxygenase activity, reducing inflammatory signaling, and limiting platelet aggregation. Furthermore, the experimental data indicate a potential role in reducing subretinal fibrosis, a key driver of visual decline in neovascular AMD [8]. Through PPAR-α activation, fibrates may also modulate anti-inflammatory and oxidative stress pathways, improve endothelial function, and enhance microvascular stability, all of which may be relevant to retinal homeostasis and angiogenic regulation in AMD [8,12,40,41]. Nevertheless, the clinical evidence supporting these mechanisms in AMD patients remains comparatively sparse, and most available support derives from preclinical or translational studies rather than direct human retinal data.

When contextualized within the contemporary clinical literature reviewed in this study, the therapeutic implications of lipid-lowering therapy remain inconclusive. The observational and secondary evidence syntheses published between 2020 and 2025 demonstrate heterogeneous findings. While several analyses suggest potential protective associations—particularly in metabolically high-risk populations—others report neutral outcomes.

For instance, Mauschitz et al. [14] observed lower AMD prevalence among patients receiving combined lipid-lowering and antidiabetic therapy. Similarly, Ganesh et al. [9] described the delayed onset of dry AMD among statin users. In contrast, other large-scale analyses have found no significant association between statin exposure and AMD risk or progression [21]. The evidence regarding fibrates remains comparatively limited, although Wang et al. [18] reported a reduced risk of dry AMD among patients demonstrating high therapeutic adherence. In such settings, the possibility of a “healthy adherer” effect should also be considered, as patients demonstrating better medication adherence may differ systematically in health behaviors, surveillance intensity, and comorbidity management compared with less adherent individuals. For this reason, the apparent protective associations observed in highly adherent users were interpreted cautiously throughout this review and were not regarded as evidence of a direct therapeutic effect. Instead, they were considered potentially influenced by a combination of adherence-related bias, more intensive medical surveillance, and better overall control of systemic comorbidities.

Importantly, the preponderance of available data does not support a statistically significant association between statin therapy and AMD incidence or progression in the general population [4,5,10,13,15]. The variability in findings likely reflects methodological heterogeneity, including differences in exposure definitions, cumulative dosing, treatment duration, and baseline cardiovascular risk profiles.

Notably, several studies have suggested that therapeutic effects—if present—may be dose-dependent or restricted to specific clinical subgroups. One analysis reported no progression to exudative AMD among patients receiving very high-dose statin therapy [10]. High-dose statin regimens—typically corresponding to high-intensity statin therapy (e.g., atorvastatin 40–80 mg or rosuvastatin 20–40 mg daily)—have been associated with a reduced AMD progression in the observational data [10], although consistent dose thresholds have not been uniformly applied across studies. Similarly, high cumulative statin exposure has been linked to a lower risk of AMD development [6]. However, these findings should be interpreted with caution due to potential confounding factors, variability in exposure definitions, and the observational nature of the available evidence.

Despite these observations, the current evidence does not justify the routine use of statins as a preventive strategy for AMD. Rather, the available literature supports a more nuanced interpretation in which potential benefits may be confined to selected populations, including individuals with an early stage of the disease, metabolic dysregulation, or prolonged high-intensity therapy exposure [13].

This review has several limitations that should be acknowledged. First, the evidence base is dominated by observational studies, which limits causal inference and increases susceptibility to residual confounding. Second, substantial heterogeneity exists across study populations, lipid-lowering regimens, AMD classification systems, and outcome definitions. Such variability complicates cross-study comparisons and precludes definitive conclusions. Third, the limited number of randomized controlled trials restricts the strength of the therapeutic recommendations.

Future research should prioritize well-designed prospective trials with standardized exposure definitions, stratification by AMD phenotype, and long-term follow-up. A particular emphasis should be placed on identifying the patient subgroups most likely to benefit from lipid modulation strategies. Elucidating the interaction between systemic lipid metabolism, genetic susceptibility, and retinal microenvironmental factors will be critical for refining preventive and therapeutic approaches in AMD.

## 5. Conclusions

The relationship between lipid-lowering therapy and age-related macular degeneration (AMD) remains an area of active investigation, yet the current clinical evidence does not establish a definitive therapeutic role for statins or fibrates in AMD prevention or disease modification.

The available studies suggest that lipid modulation may influence retinal pathology through mechanisms related to cholesterol metabolism, inflammation, and vascular function. However, clinical findings are heterogeneous and largely derived from observational analyses, limiting causal interpretation. While several investigations report delayed disease onset or slower progression among statin users, these associations appear to be context-dependent rather than generalizable across populations.

A potential benefit has been most frequently observed in patients with early or non-exudative AMD, as well as in individuals exposed to long-term or high-intensity statin therapy. This may reflect the greater modifiability of lipid-related and inflammatory pathways during earlier stages of disease than in advanced atrophic or neovascular phenotypes. In contrast, the evidence regarding fibrates remains comparatively sparse. The existing data suggest that therapeutic adherence and cumulative exposure may be relevant to risk modulation; however, any inference regarding the clinical benefit remains tentative given the small number of available studies, their heterogeneity, and the predominantly observational nature of the evidence.

At present, the available literature does not support the routine use of lipid-lowering agents as a universal preventive or therapeutic strategy in AMD. Nevertheless, the biological plausibility of lipid involvement in retinal degeneration, together with the subgroup signals observed in clinical studies, underscores the need for further targeted investigation.

The main gaps in the current evidence base include the predominance of observational designs, inconsistent exposure definitions, limited phenotype-specific analyses, and insufficient data on treatment duration, cumulative dose, and metabolic risk stratification. Future research should prioritize prospective trials with standardized exposure definitions, clearer stratification by AMD phenotype, and more precise characterization of metabolic risk profiles. Such approaches may help identify the patient subgroups most likely to benefit from lipid-lowering therapy and clarify whether observed therapeutic signals reflect true biological effects or residual confounding.

## Figures and Tables

**Table 1:** The key clinical studies and evidence syntheses evaluating lipid-lowering therapy and age-related macular degeneration (2020–2025). Abbreviations: AMD, age-related macular degeneration; N/A, not applicable; USA, United States of America.

Author	Year	Country	Study Design	Population	Therapy Evaluated	Exposure Definition/AMD Subtype	Key Outcomes Assessed	Main Findings
Ludwig [10]	2021	USA	Cohort	231,888	Statins and Fibrates	Statin/fibrate exposure; exudative AMD progression	Progression to exudative AMD	No association with exudative AMD progression
Wang [18]	2021	Taiwan	Cohort	68,751	Fibrates	fibrate adherence-based exposure; dry AMD	Dry AMD incidence	Reduced dry AMD risk in adherent users
Memarzadeh [15]	2022	Iran	Systematic Review & Meta-Analysis	2,063,195	Statins	statin exposure (any use); any AMD risk	Risk of AMD	No significant association with AMD risk
Eshtiaghi [5]	2022	Canada	Meta-Analysis	1,460,989	Statins	statin exposure (any vs non-use); AMD incidence/progression	AMD incidence and progression	No significant difference in AMD incidence or progression
Grimes [8]	2023	USA	Review Article	N/A	Fibrates	statin exposure (cumulative/duration-based); AMD progression	AMD-related inflammation and fibrosis	Preclinical protective effects suggested
Lymperopoulou [13]	2023	Cyprus, Greece	Systematic Review	28,940	Statins	statin exposure (cumulative/duration-based); AMD progression	AMD progression	Inconclusive evidence of protective effects
Mauschitz [14]	2023	Europe	Meta-Analysis	38,694	Statins and Fibrates	lipid-lowering drugs overall exposure; any/late AMD prevalence	Prevalent any and late AMD	Lower prevalence of any AMD, no association with late AMD
Chen [6]	2023	Taiwan	Cohort	92,892	Statins and Fibrates	statin exposure by dose category (high vs low dose); AMD risk/progression	AMD risk and progression	Dose-dependent observational association; possible confounding
Ganesh [9]	2023	USA	Cohort	62,817	Statins	statin exposure (any use); onset of AMD	Onset of dry AMD	Later onset in dry AMD
Shaw [21]	2024	USA	Case-Control	160,759	Statins	statin exposure (any vs non-use); AMD development	AMD development	No significant association with AMD development
Dascalu [4]	2024	Romania	Systematic Review	1,268,741	Statins	statin exposure (dose-dependent/cumulative); AMD progression/visual acuity	AMD progression and visual acuity	High-dose benefit suggested in AMD with soft indistinct drusen

## Data Availability

Data sharing does not apply to this article as no new data were created or analyzed in this study.

## Abbreviations

The following abbreviations are used in this manuscript:

AMD	Age-related macular degeneration
BrM	Bruch’s membrane
HMG-CoA	3-hydroxy-3-methylglutaryl-coenzyme A
PPAR-α	Peroxisome proliferator-activated receptor alpha
RPE	Retinal pigment epithelium
VEGF	Vascular endothelial growth factor
